# Alarming low adherence to the 24-Hour Movement Behavior in adolescents — the Cogni-Action Project

**DOI:** 10.3389/fpubh.2025.1646147

**Published:** 2025-09-04

**Authors:** Luis Felipe Rojas-Araya, Juan Pablo Espinoza-Puelles, Gerson Ferrari, Marcelo Toledo-Vargas, Nicolas Aguilar-Farias, Carlos Cristi-Montero

**Affiliations:** ^1^Departamento de Ciencias Biomédicas, Universidad de León, León, Spain; ^2^Carrera de Pedagogía en Educación Física, Facultad de Educación, Universidad Autónoma de Chile, Talca, Chile; ^3^Center for Social and Cognitive Neuroscience (CSCN), School of Psychology, Universidad Adolfo Ibáñez, Santiago, Chile; ^4^IRyS Group, Physical Education School, Pontificia Universidad Católica de Valparaíso, Viña del Mar, Chile; ^5^Universidad de Santiago de Chile (USACH), Escuela de Ciencias de la Actividad Física, el Deporte y la Salud, Santiago, Chile; ^6^Facultad de Ciencias de la Salud, Universidad Autónoma de Chile, Providencia, Chile; ^7^Department of Physical Education, Sports and Recreation, Universidad de La Frontera, Temuco, Chile; ^8^School of Social Sciences, Faculty of the Arts, Social Sciences and Humanities, University of Wollongong, Wollongong, NSW, Australia

**Keywords:** 24-hour activity cycle, exercise, sleep quality, sedentary behavior, wearables

## Abstract

**Background:**

Unhealthy movement behaviors are a critical concern, particularly in developing countries and regions like Latin America. Evaluating adherence to the 24-Hour Movement Behavior (24-HMB) through a combination of objective and self-reported measures of moderate-to-vigorous physical activity (MVPA), adequate sleep duration (SD), and limited sedentary time (ST) is essential for understanding the interplay among these behaviors during adolescence.

**Objective:**

This cross-sectional study aimed to assess adherence to the 24-HMB among Chilean adolescents aged 10–14 years and explore differences by age and sex.

**Methods:**

A total of 359 participants (53.48% girls) wore accelerometers to objectively measure compliance with the recommended 60 min of MVPA per day, as well as appropriate SD (9–11 h for ages 10–13 and 8–10 h for age 14). ST was assessed through self-reported data (≤2 h/day).

**Results:**

Compliance with the MVPA, SD, and ST recommendations was achieved by 0.84, 4.74, and 70.47% of participants, respectively. When combining guidelines, only 0.56% of participants met both the ST and MVPA recommendations, while 3.90% adhered to both the ST and SD recommendations. Notably, no participants met both the SD and MVPA guidelines, nor did any fulfill all three movement guidelines. Finally, no significant differences were observed by sex or age.

**Conclusion:**

These findings underscore the alarmingly low adherence to the 24-HMB among this sample of adolescents, highlighting the urgent need for targeted public health interventions. The study advocates for policymakers to adopt an integrated approach to promote healthy behaviors, addressing them collectively rather than in isolation.

## Introduction

South American adolescents’ lifestyle behaviors often coexist negatively, particularly regarding movement behaviors, with a notable impact on girls and a tendency to worsen with age (1). In Chile, this pattern is reflected in the findings of the most recent National Survey on Physical Activity and Sport, which reported that only 3 out of 10 children and adolescents aged 5 to 17 years meet the minimum physical activity recommendation (2). The 24-Hour Movement Behavior (24-HMB) provides an evidence-based framework that integrates moderate-to-vigorous physical activity (MVPA), adequate sleep duration (SD), and limited sedentary time (ST) to promote healthy lifestyles and optimize health outcomes in this population (3, 4). It is essential to examine 24-HMB in an integrated manner to fully understand its cumulative impact on adolescent health. Numerous studies have demonstrated that these behaviors do not operate independently; rather, they interact with each other, jointly influencing physical, mental, and cognitive well-being. In this context, a recent meta-analysis (5) revealed that adolescents who simultaneously meet all three recommendations of the 24-HMB framework exhibit more favorable levels of adiposity, cardiometabolic health, psychological well-being, academic performance, and quality of life compared to their peers who do not. These findings reinforce the importance of evaluating these behaviors collectively rather than in isolation, particularly in settings where adherence rates are low (5–7). Adherence to these guidelines is consistently linked to benefits such as reduced adiposity, improved fitness, enhanced well-being, better quality of life, and superior cognitive performance (6).

Supporting this concern, a recent study involving 10,574 children aged 9–11 years found that only 49% met sleep duration guidelines, 35% met screen time recommendations, and only 17% met physical activity guidelines. Alarmingly, only 4% of the participants met all three 24-HMB recommendations, whereas 31% did not meet any (8). These results highlight the global challenge of promoting healthy movement behaviors among youth and reinforce the need for objective, context-specific data.

A recent meta-analysis of over 387,000 youth aged preschool to adolescence across 23 countries found that only 7.12% met all three components of the movement guidelines. In South America, adherence was even lower, with only 2.93% of youth meeting the guidelines, and compliance was positively correlated with each country’s Human Development Index (9). These findings highlight a significant gap in adherence to essential health guidelines, particularly in developing countries. Moreover, much of the evidence relies on self-reported data from Latin American adolescents, which is prone to bias and inaccuracies (7, 10). Moreover, significant methodological limitations persist, such as the use of self-reported data, cross-sectional study designs, and the lack of standardized measurement protocols, all of which may compromise the accuracy of the findings of these studies. In this context, the present study provides more robust evidence by objectively assessing physical activity and sleep through accelerometry, thereby reducing bias and enhancing data quality (5, 6, 9).

Chile, a nation undergoing epidemiological and socioeconomic transitions, provides a unique context for examining these behaviors. Assessing adherence to the three movement-related health guidelines during adolescence (7, 11) offers valuable insights for shaping policies and educational interventions tailored to the realities of developing countries. This study aimed to determine adherence to the 24-HMB among Chilean adolescents and to explore potential age and sex differences.

## Methodology

This cross-sectional study, part of the Cogni-Action Project (12), was conducted between March 2017 and October 2019, following the STROBE guidelines and was approved by the Bioethics Committee of the Pontificia Universidad Católica de Valparaíso, Chile. Informed consent was obtained from school principals, parents, and participants. The project initially included 1,296 adolescents (50% girls) aged 10 to 14 years. A subsample of 436 schoolchildren, determined through power estimation, was instructed to wear an accelerometer (12). For this subsample, an appropriate sample size of 436 participants was estimated, based on a 5% alpha error, a 99% confidence interval, 50% heterogeneity, and an expected 20% dropout rate (12). After excluding participants without valid accelerometry data, the final sample included 359 adolescents.

### 24-hour movement guidelines

Participants were evaluated at school during two visits, eight days apart, conducted by trained instructors. During the first visit, sociodemographic characteristics and body composition were assessed (Table 1). Participants were also provided with accelerometers to evaluate their MVPA and SD. During the second visit, trained professionals administered a standardized questionnaire to evaluate ST. Accelerometers were collected at the end of the second visit.

**Table 1:** Descriptive characteristics of the participants and prevalence of the three 24-HMB.

Variables	Overall (*n* = 359)	Boys (*n* = 167)	Girls (*n* = 192)	*p*-value
Age (years)	12.0 ± 1.1	11.9 ± 1.1	12.1 ± 1.1	0.314
Weight (kg)	50.2 ± 11.7	50.1 ± 12.5	50.4 ± 11	0.825
Height (cm)	153 ± 9.1	154 ± 10.5	153 ± 7.7	0.171
Waist (cm)	68.4 ± 8.6	69.6 ± 8.7	67.3 ± 8.3	**0.010**
Body mass index (kg/m^2^)	21.2 ± 3.7	20.9 ± 3.6	21.4 ± 3.7	0.156
Waist-to-height ratio	0.45 ± 0.05	0.45 ± 0.05	0.44 ± 0.05	**0.041**
Meeting recommendations				
Only MVPA	3 (0.84%)	3 (1.8%)	0 (0.0%)	0.062
Only Sleep duration	17 (4.74%)	11 (6.59%)	6 (3.13%)	0.123
Only Screen time	253 (70.47%)	111 (66.47%)	142 (73.96%)	0.121
MVPA + Sleep duration	0 (0.0%)	0 (0.0%)	0 (0.0%)	N/A
MVPA + Screen time	2 (0.56%)	2 (1.2%)	0 (0.0%)	0.128
Sleep + Screen time	14 (3.9%)	10 (5.99%)	4 (2.08%)	0.057
All Three	0 (0.0%)	0 (0.0%)	0 (0.0%)	N/A

Table 1 provides demographic data by sex and associated *p*-values, with significant values in bold, calculated using the Student’s t-test for continuous variables and the chi-square test for categorical variables. Data are expressed as mean ± standard deviation or frequency (percentage). MVPA: moderate vigorous physical activity; SD: sleep duration; ST: screen time; N/A: insufficient data for comparison.

MVPA and SD were assessed using an accelerometer (ActiGraph GT3X, Pensacola, USA) worn on the right hip for 7 days. Raw acceleration data (100 Hz) were processed in R using the GGIR package version 3.0–2 (13). MVPA was defined using hip-based cut-off points for the Euclidean Norm Minus One (ENMO) metric (14), and SD was estimated using the HorAngle algorithm for hip-worn devices implemented in GGIR (15). Adolescents who wore the device for at least 16 h per day on at least 4 days per week, including a minimum of three weekdays and one weekend day, were considered to have valid accelerometry recordings and were included in the analysis (359 adolescents) (14).

Participants met the PA and SD guidelines if they accumulated at least 60 min of MVPA per day and achieved 9–11 h of SD for ages 10–13 or 8–10 h for age 14, respectively (3, 4, 16). They were categorized as meeting the ST guidelines if they spent ≤2 h per day on sedentary recreational screen time (3, 4, 16). ST was assessed using the Godard questionnaire, which accounts for time spent watching television, using a computer, and playing video games (17).

### Statistical analysis

**Figure 1:**
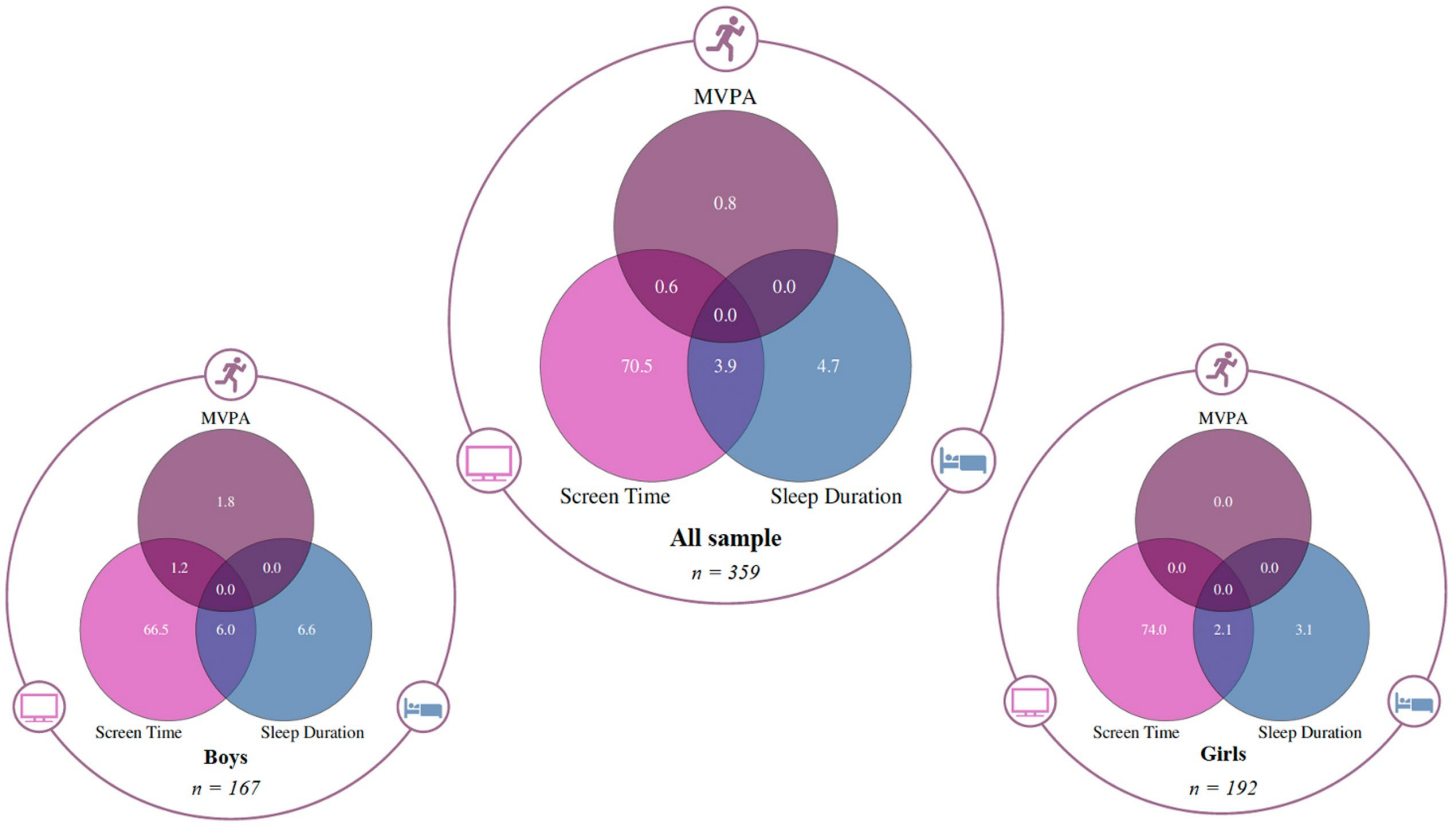
Venn diagrams illustrating the proportion (%) of boys, girls, and all participants meeting 24-HMB. MVPA: moderate-to-vigorous physical activity, ST: screen time, SD: sleep duration, and all possible combinations.

Continuous variables were reported as means or medians, and categorical variables as percentages. Proportions meeting the criteria by sex and age were compared using the chi-square test. Compliance proportions are displayed in a Venn diagram (Figure 1). Statistical significance was set at *p* ≤ 0.05.

## Results

Descriptive characteristics and adherence to the 24-HMB guidelines are presented in Table 1. Of the 359 adolescents included in the study, 53.48% were girls. Only 0.84% met the MVPA recommendation, 4.74% met the SD guideline, and 70.47% adhered to the ST recommendation. None of the participants met all three guidelines simultaneously, nor did any meet both MVPA and SD. Only 0.56% met both MVPA and ST, and 3.90% met both SD and ST. Moreover, no significant sex-based differences were observed in adherence to MVPA (*p* = 0.062), SD (*p* = 0.123), ST (*p* = 0.121), MVPA + ST (*p* = 0.128), or SD + ST (*p* = 0.057). Age-specific analysis showed that none of the adolescents aged 10, 12, or 14 years met the MVPA guideline, while one 11-year-old and two 13-year-olds did. The highest compliance rates for SD and ST were observed at ages 14 (25.9%) and 11 (83.0%), respectively (Table 2).

**Table 2:** Adherence to the three 24-Hour Movement Behavior components and their combinations, reported by age.

Percentage of meeting 24-HMB according to age								
Age subgroups	*n* (%)	Only MVPA%	Only SD %	Only ST %	MVPA+ SD %	MVPA+ ST %	SD+ ST %	All Three %
10	45 (12.53)	0 (0.0)	1 (2.22)	35 (77.78)	0 (0.0)	0 (0.0)	1 (2.22)	0 (0.0)
11	53 (14.76)	1 (1.89)	3 (5.66)	44 (83.02)	0 (0.0)	1 (1.89)	3 (5.66)	0 (0.0)
12	144 (40.11)	0 (0.0)	5 (3.47)	95 (65.97)	0 (0.0)	0 (0.0)	4 (2.78)	0 (0.0)
13	90 (25.07)	2 (2.22)	1 (1.11)	61 (67.78)	0 (0.0)	1 (1.11)	1 (1.11)	0 (0.0)
14	27 (7.52)	0 (0.0)	7 (25.93)	18 (66.67)	0 (0.0)	0 (0.0)	5 (18.52)	0 (0.0)
All age	359 (100.0)	3 (0.84)	17 (4.74)	253 (70.47)	0 (0.0)	2 (0.56)	14 (3.9)	0 (0.0)

Table 2 summary of adherence to the 24-Hour Movement Behavior components by age group.

As shown in Figure 1, the Venn diagram visually illustrates the limited overlap between the three behaviors. Most participants met only the ST guideline, while very few met combinations of two behaviors, and none met all three. This visual representation reinforces the low overall adherence to the integrated 24-HMB guidelines and highlights the need for targeted interventions to promote balanced movement behaviors among adolescents.

## Discussion

The results of this study revealed alarmingly low adherence to the 24-HMB among Chilean adolescents, consistent with global findings, particularly in South America, where low adherence rates are prevalent (9, 10, 18, 19). Furthermore, this trend appears to extend across both boys and girls, regardless of age.

Addressing adherence to the 24-HMB requires an integrated approach that considers the recommendations as interconnected components rather than isolated targets (3, 9, 20). Physical activity, sedentary behavior, and sleep are intrinsically linked, and interventions should prioritize strategies that promote a balanced daily routine (10, 20, 21). For instance, excessive screen time may not only reduce physical activity but also disrupt sleep patterns, amplifying the negative health outcomes (20, 22). This highlights the importance of designing comprehensive interventions that simultaneously address all three recommendations, ensuring they complement rather than compete with one another (23).

This study provides evidence of low adherence to the guidelines across all demographic subgroups, with no significant differences observed by sex or age. This uniformity suggests that interventions should target the entire adolescent population rather than focusing on specific subgroups. Furthermore, the findings suggest that systemic and cultural barriers may be influencing adherence at a population level.

Contextual factors may help explain these low adherence rates. Chilean adolescents report low levels of active transport and social support, alongside socioeconomic challenges such as income inequality and child poverty (18). These structural barriers likely constrain the adoption of healthy movement behaviors, underscoring the importance of context-sensitive public health strategies. In light of these findings, we propose three actionable recommendations: incorporating daily physical education into the school curriculum to ensure equitable access to physical activity; regulating screen time through coordinated school-family partnerships that promote consistent routines; and implementing sleep hygiene education programs targeting both adolescents and their caregivers. These strategies, grounded in the local context, may help foster healthier 24-h movement patterns and reduce disparities in health outcomes.

### Strengths and limitations

This study stands out for its use of objective accelerometry to assess MVPA and SD, combined with validated self-report for ST, offering a comprehensive evaluation of 24-HMB adherence in Chilean adolescents. However, its cross-sectional design limits causal interpretations. ST was self-reported, which may introduce bias. The regional sample may limit generalizability.

## Conclusion

The alarmingly low adherence to the 24-HMB among Chilean adolescents underscores the urgent need for targeted policies and programs, particularly to increase MVPA and SD. Multifaceted interventions involving schools, families, and communities are essential to effectively address these gaps. This situation highlights the importance of public health efforts that focus on both movement-related and non-movement-related behaviors, fostering a more holistic approach to adolescent health.

## Data Availability

The data supporting the conclusions of this article will be made available by the authors, without undue reservation.

## References

[1] Matias TS de Oliveira Araujo RH Tassitano RM Ramírez-Vélez R Sadarangani KP Ywgne J. Clustering of obesogenic behaviours amongst 140 052 south American adolescents: a harmonized meta-analysis of national health surveys. J Public Health. (2025):fdae 319. doi: 10.1093/pubmed/fdae319, PMID: 39827086

[2] Ligup. Ministerio del Deporte. Resultados de la Encuesta Nacional de Actividad Física y Deporte 2024. Available online at: https://www.mindep.cl/actividades/noticias/2426

[3] Tremblay MS Carson V Chaput JP Connor Gorber S Dinh T Duggan M. Canadian 24-hour movement guidelines for children and youth: an integration of physical activity, sedentary behaviour, and sleep. Appl Physiol Nutr Metab. (2016) 41:S311–27. doi: 10.1139/apnm-2016-0151, PMID: 27306437

[4] Okely AD Ghersi D Loughran SP Cliff DP Shilton T Jones RA. A collaborative approach to adopting/adapting guidelines. The Australian 24-hour movement guidelines for children (5-12 years) and young people (13-17 years): an integration of physical activity, sedentary behaviour, and sleep. Int J Behav Nutr Phys Act. (2022) 19:2. doi: 10.1186/s12966-021-01236-2, PMID: 34991606 PMC8734238

[5] Zhao H Wu N Haapala EA Gao Y. Association between meeting 24-h movement guidelines and health in children and adolescents aged 5–17 years: a systematic review and meta-analysis. Front Public Health. (2024) 12:1351972. doi: 10.3389/fpubh.2024.1351972, PMID: 38774055 PMC11106490

[6] Rollo S Antsygina O Tremblay MS. The whole day matters: understanding 24-hour movement guideline adherence and relationships with health indicators across the lifespan. J Sport Health Sci. (2020) 9:493–510. doi: 10.1016/j.jshs.2020.07.004, PMID: 32711156 PMC7749249

[7] Guedes DP Zuppa MA. Adherence to combined healthy movement behavior guidelines among adolescents: effects on Cardiometabolic health markers. Int J Environ Res Public Health. (2022) 19:8798. doi: 10.3390/ijerph19148798, PMID: 35886650 PMC9319843

[8] Fung H Yeo BTT Chen C Lo JC Chee MWL Ong JL. Adherence to 24-hour movement recommendations and health indicators in early adolescence: cross-sectional and longitudinal associations in the adolescent brain cognitive development study. J Adolesc Health. (2023) 72:460–70. doi: 10.1016/j.jadohealth.2022.10.019, PMID: 36528521

[9] Tapia-Serrano MA Sevil-Serrano J Sánchez-Miguel PA López-Gil JF Tremblay MS García-Hermoso A. Prevalence of meeting 24-hour movement guidelines from pre-school to adolescence: a systematic review and meta-analysis including 387, 437 participants and 23 countries. J Sport Health Sci. (2022) 11:427–37. doi: 10.1016/j.jshs.2022.01.005, PMID: 35066216 PMC9338333

[10] Da Costa BGG Chaput JP Lopes MVV Malheiros LEA Tremblay MS Silva KS. Prevalence and sociodemographic factors associated with meeting the 24-hour movement guidelines in a sample of Brazilian adolescents Paul D, editor. PLoS One. (2020) 15:e0239833. doi: 10.1371/journal.pone.0239833, PMID: 32986765 PMC7521749

[11] van Sluijs EMF Ekelund U Crochemore-Silva I Guthold R Ha A Lubans D. Physical activity behaviours in adolescence: current evidence and opportunities for intervention. Lancet. (2021) 398:429–42. doi: 10.1016/S0140-6736(21)01259-9, PMID: 34302767 PMC7612669

[12] Solis-Urra P Olivares-Arancibia J Suarez-Cadenas E Sanchez-Martinez J Rodríguez-Rodríguez F Ortega FB. Study protocol and rationale of the “Cogni-action project” a cross-sectional and randomized controlled trial about physical activity, brain health, cognition, and educational achievement in schoolchildren. BMC Pediatr. (2019) 19:260. doi: 10.1186/s12887-019-1639-8, PMID: 31349791 PMC6659252

[13] Migueles JH Rowlands AV Huber F Sabia S van Hees VT. GGIR: a research community–driven open-source R package for generating physical activity and sleep outcomes from multi-day raw accelerometer data. J Measure Physical Behav. (2019) 2:188–96. doi: 10.1123/jmpb.2018-0063

[14] Migueles JH Cadenas-Sanchez C Ekelund U Delisle Nyström C Mora-Gonzalez J Löf M. Accelerometer data collection and processing criteria to assess physical activity and other outcomes: a systematic review and practical considerations. Sports Med. (2017) 47:1821–45. doi: 10.1007/s40279-017-0716-0, PMID: 28303543 PMC6231536

[15] van Hees VT Sabia S Jones SE Wood AR Anderson KN Kivimäki M. Estimating sleep parameters using an accelerometer without sleep diary. Sci Rep. (2018) 8:12975. doi: 10.1038/s41598-018-31266-z, PMID: 30154500 PMC6113241

[16] J I R KC T W. Adherence to the 24-hour movement guidelines among 10-to 17-year-old Canadians. Health Promot Chronic Dis Prev Can Res Policy Pract. (2017) 37:369–75. doi: 10.24095/hpcdp.37.11.01 PMC5695900 29119774

[17] Godard MC del P RNM Díaz N Lera ML Salazar RG Burrows AR. Valor de un test clínico para evaluar actividad física en niños. Rev Médica Chile. (2008) 136:1155–62. doi: 10.4067/S0034-98872008000900010 19030660

[18] Toledo-Vargas M Perez-Contreras P Chandia-Poblete D Aguilar-Farias N. Compliance of the 24-hour movement guidelines in 9-to 11-year-old children from a low-income town in Chile. J Phys Act Health. (2020) 17:1034–41. doi: 10.1123/jpah.2019-0672, PMID: 32866944

[19] Fan H Fan G Meng J. Association between family factors and 24-h movement behaviors of adolescents—a cross-sectional study of Chinese high school students. Front Public Health. (2025) 13:1564423. doi: 10.3389/fpubh.2025.1564423, PMID: 40458108 PMC12127378

[20] Pedersen J Rasmussen MGB Sørensen SO Mortensen SR Olesen LG Brønd JC. Effects of limiting recreational screen media use on physical activity and sleep in families with children: a cluster randomized clinical trial. JAMA Pediatr. (2022) 176:741–9. doi: 10.1001/jamapediatrics.2022.1519, PMID: 35604678 PMC9127712

[21] García-Hermoso A Ezzatvar Y López-Gil JF. Association between daily physical education attendance and meeting 24-hour movement guidelines in adolescence and adulthood. J Adolesc Health. (2023) 73:896–902. doi: 10.1016/j.jadohealth.2023.06.014, PMID: 37610389

[22] Baranwal N Yu PK Siegel NS. Sleep physiology, pathophysiology, and sleep hygiene. Prog Cardiovasc Dis. (2023) 77:59–69. doi: 10.1016/j.pcad.2023.02.005, PMID: 36841492

[23] Bergmann GG Gaya AR Silva LR Cruz JH de B da Motta TC Ferreira GD. 24-hour movement guidelines: descriptive study with overweight and obese low-income children. J Mov Health (2023) 20:1–11. doi: 10.5027/jmh-Vol20-Issue2(2023)art183

